# The Burden of Vertebral Osteomyelitis—An Analysis of the Workforce before and after Treatment

**DOI:** 10.3390/jcm11041095

**Published:** 2022-02-18

**Authors:** Ayla Yagdiran, Jan Bredow, Carolyn Weber, Ghaith Mousa Basha, Peer Eysel, Julia Fischer, Norma Jung

**Affiliations:** 1 Department of Orthopedic and Trauma Surgery, Faculty of Medicine and University Hospital of Cologne, University of Cologne, 50923 Cologne, Germany; ghaith.mousabasha@yahoo.com (G.M.B.); peer.eysel@uk-koeln.de (P.E.); 2 Department of Orthopedic and Trauma Surgery, Krankenhaus Porz am Rhein, 51149 Cologne, Germany; j.bredow@khporz.de; 3 Heart Center, Department of Cardiothoracic Surgery, Faculty of Medicine and University Hospital of Cologne, University of Cologne, 50923 Cologne, Germany; carolyn.weber@uk-koeln.de; 4 Center for Integrated Oncology, Department I of Internal Medicine, Faculty of Medicine and University Hospital of Cologne, University of Cologne, 50923 Cologne, Germany; julia.fischer@uk-koeln.de (J.F.); norma.jung@uk-koeln.de (N.J.); 5 Center for Molecular Medicine Cologne (CMMC), University of Cologne, 50931 Cologne, Germany; 6 German Center for Infection Research (DZIF), Partner Site Bonn-Cologne, 50935 Cologne, Germany

**Keywords:** spondylodiscitis, workforce, ability to work, return to work, disability pension

## Abstract

Although vertebral osteomyelitis (VO) has a major impact on morbidity, functional status, and quality of life, data concerning the influence on the patient’s ability to work (ATW) are lacking. Therefore, the aim of this study was to analyze the work status after VO-treatment as well as risk factors associated with loss of the ATW. We conducted a post-hoc analysis of data from a prospective VO-registry (2008–2019) supplemented by workforce data. Primary endpoint was the work status after one year (T1). Univariate analysis comparing patients’ characteristics “at-work” versus “not-at-work” at T1 was performed. Of a total of 335 VO-patients, *n* = 52 (16%) were part of the workforce at time of diagnosis (T0), of which 22 (42%) failed to be part of the workforce at T1. A higher number of comorbidities and a body mass index (BMI) < 25 kg/m^2^ were associated with a reduced ATW. VO in working age patients is a debilitating condition and associated with reduced patients’ ATW. Patients engaged in heavy physical work mostly had a BMI < 25 kg/m^2^ and therefore were more severely affected and no longer able to keep their workforce. More support in retraining should be offered after successful treatment to maintain ATW and reduce the socio-economic burden.

## 1. Introduction

Despite the rising incidence in recent years, vertebral osteomyelitis (VO) remains a rare disease. However, VO is a severe condition often associated with a complicated clinical course of illness [1,2,3]. Moreover, the overall mortality has been reported up to 20% and appears to be particularly high in the first year after diagnosis [2,4,5,6]. The principal treatment for VO is targeted antibiotic therapy with or without surgery [3,7]. Previous studies have described the prognosis among patients with VO in terms of recurrence, mortality, functional outcome, and quality of life (QoL) [2,4,5,6,8,9]. A systematic review showed that neurological deficit and recurrence occur in one third of cases after VO, respectively [10]. Thus, VO is frequently causing a profound impact on long-lasting back pain, function and QoL resulting in an increased short- and long-term mortality [2,3,4,5,10,11,12]. Many of these patients never regain full spinal function, QoL levels remain below those of the normal population as well as their work ability suggesting a significant socio-economic burden for society [6,13].

Although more than 30% of the VO patients are among working-age there is only limited data of how VO affects the ability to work (ATW) [13]. Especially, it remains elusive how the profession and work-time is affected. Thus, the aim of this study was to assess the ATW, risk for sick leave, disability pension and mortality in a cohort of VO patients within one year after treatment.

## 2. Materials and Methods

### 2.1. Patient Selection

For this study, we used prospectively collected data from 2008 until 2019 from the former European “Spine Tango” now “Deutsches Wirbelsäulen Gesellschaft (DWG)” register. Patients were diagnosed with VO at the Department for Orthopaedics and Trauma at a tertiary referral hospital based on the presence of characteristic back and/or leg pain plus characteristic magnetic resonance imaging (MRI) or an abscess or vertebral body destruction detected by computed tomography (CT). All cases were discussed in an interdisciplinary manner between an infectious disease specialist and an orthopaedic surgeon to confirm the diagnosis of VO. Adult patients between 18 and 63 years of age were considered for analysis. As retirement pension in Germany is available from 65 years of age, we chose to include only those aged ≤63 years, allowing one year of follow up before the possibility of retirement pension.

### 2.2. Data Collection

The following data were prospectively collected after enrollment: age, sex, length of hospital stay, affected spine segment and American Society of Anesthesiologists (ASA) score. The ASA Physical Status Classification System was developed in 1941 to classify patient comorbidity and is widely used by clinicians. ASA Class I is defined as a normal healthy patient, and Class V as a moribund patient not expected to survive without surgery. In addition, the following demographic and clinical parameters were recorded for all VO patients: bacteremia, causative pathogens, body mass index (BMI), relevant comorbidities (diabetes, oncologic disease, chronic obstructive pulmonary disease = COPD, inflammatory bowel disease = IBD, rheumatic disease, cardiac insufficiency, renal insufficiency, endocarditis, alcohol and drug abuse), laboratory, Charlson comorbidity index (CCI), osseous destruction of the vertebrae based on Eysel/Peters classification for spondylodiscitis, presence of psoas abscess or empyema and pre-operative neurological deficits based on the Frankel scale. The Frankel Scale classifies the extent of the neurological/functional deficit into five grades. Grade A shows no motor or sensory function below the level of lesion, whereas Grade E is defined as a normal motor and/or sensory function. The CCI was developed in 1987 as a weighted index to predict risk of death within 1 year of hospitalization for patients with specific comorbid conditions. The CCI score can be categorized into three degrees: mild (CCI 1–2), moderate (CCI 3–4) and severe (CCI ≥ 5). The Eysel/Peters classification categorizes the vertebral destruction into 4 grades. Grade I is defined as a decrease of the intervertebral space in contrast to Grade IV defined as a reactive bone formation in the sense of a support reaction and incipient kyphotic malalignement. The classification of the BMI was based on the WHO definition. Underweight is defined as a BMI ≤ 19, overweight is a BMI ≥ 25, and a BMI of >30 kg/m^2^ is defined as obesity.

### 2.3. Assessment of ATW

To assess ATW a follow-up survey one year after VO treatment (T1) was conducted. All patients were contacted and interviewed by telephone. In case of a missing response, questionnaires were sent by mail. The following parameters were collected: type of work and work time (full-/part-time) at T0 and T1, respectively and status pension. In addition, the patients‘ satisfaction on their VO treatment was evaluated on a scale of 1–4 points. This simple survey is a subjective measurement for a roughly grading of satisfaction after VO treatment.

1. Not satisfied with treatment results after VO treatment.
2. Slightly satisfied with treatment results after VO treatment.
3. Moderately satisfied with treatment results after VO treatment.
4. Fully satisfied with treatment results after VO treatment.

ATW was defined as either working or being ‘employable’, without having a permanent ‘certificate of disability’ (“Arbeitsunfähigkeitsbescheinigung”) thus not receiving any kind of pension and leaving permanently the workforce, respectively.

Additionally, the type of work was divided into heavy and light physical work. Accordingly, the following professions were assigned to these two categories.

Heavy: Carpenter, Printing House Employee, Scaffolder, Car mechanic, Lead worker, Farrier, Mechanical Engineering, Production Worker, Welder, Auto electrician, Electrician, Construction worker, Luggage service, Hairdresser, Police officer, Bus driver, Cleaner, Facility staff.

Light: Opera singer, Manager, Network administrator, Lawyer, Agency employee, Medical assistant, Journalist, Office employee, Bank employee, IT coordinator, Purchase manager, Setter, Realtor, Management Consultant, Car salesman, Interior decorator.

### 2.4. Definition of Endpoints

Primary endpoint was work status at 12 months (T1) after VO treatment. ATW was defined as either working or being ’employable´, without having a permanent ‘certificate of disability’ (“Arbeitsunfähigkeitsbescheinigung”) thus not receiving any kind of pension and leaving permanently the workforce, respectively.

### 2.5. Statistical Analysis

All statistical analyses were performed using IBM SPSS Statistics Version 26 (IBM, Armonk, NY, USA). Unless otherwise indicated, continuous variables are described using mean values ± standard deviation or median (interquartile range) according to the normality of their distribution and compared using unpaired *t* test or Mann–Whitney U test as appropriate. Discrete variables are reported as percentages and tested by Pearson chi-square test or, when validity conditions were not satisfied, by Fisher’s exact test. Missing data were not imputed and were assumed to be missing at random. Potential risk factors for the ability to work after one year were assessed using logistic regression. All reported *p* values are 2-sided.

## 3. Results

**Figure 1:**
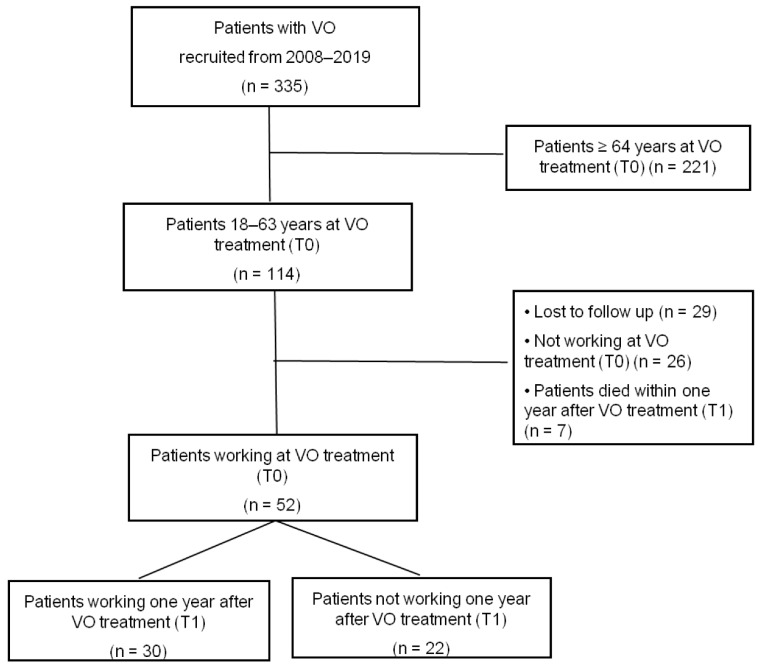
Flowchart for patient selection, VO = vertebral osteomyelitis.

In total, 353 patients with confirmed VO were enrolled in the register, 114 of whom were aged between 18 and 63 years at the time of treatment (T0). Eleven of them (10%) were on early retirement and 15 (13%) were not part of the active workforce at T0. 29 (25%) patients were lost to follow-up and during the follow-up, 7 of 114 cases died within the first year. This yields a 1-year mortality rate of 6%. Thus, 52 (46%) cases were included in the time to ATW analyses (see details in Figure 1).

### 3.1. Baseline Characteristics

The 52 analyzed VO patients had a median age of 55 years, and the majority of the patients were male (81%); 71% of the analyzed cohort were overweight/obese. The lumbar spine was most commonly affected (71%); 29% of the patients had severe comorbidities (ASA score ≥ 3). Microbiological diagnosis was established in 40 patients (77%). *Staphylococcus aureus (S. aureus)* was the most commonly isolated pathogen (*n* = 19/52; 37%). The recurrence rate was 12%. Neurologic impairment at diagnosis occurred in 6 patients (12%). The demographics and clinical characteristics are shown in Table 1.

One year after VO treatment (T1), 30 (58%) patients were able to work. These patients had less comorbidities (<2), did not suffer from a rheumatic disease and had a higher rate of overweight/obesity BMI (67% vs. 55%). Patients not working tended to have a higher CCI (2) and a chronic kidney disease (9%); additionally, those patients more often were operated (96%).

### 3.2. Patients’ Satisfaction

At T1, 67% of the working patients were fully satisfied with treatment results after VO. None of the working VO patients was not satisfied with treatment results after VO. Among the not-working patients, 18% were each not satisfied or slightly satisfied with their treatment results after VO. 36% of the not working VO patients were fully satisfied with treatment results after VO.

### 3.3. Workforce

The follow-up results of the individual workforce are listed in Table 2. Out of 52 analyzed VO patients 22 (42%) were not able to work at all one year after treatment (T1). Out of these patients, 13 (25%) received disability pension and 9 (17%) were on sick leave at T1. At T0 and T1 38 (73%) and 16 (31%) patients worked full-time, respectively. Within the 30 patients who were able to work at T1, 5 patients changed their former profession. Before VO treatment, more than half of the patients (56%) were engaged in heavy physical work. One year after VO treatment, only 41% (12/29) of those patients were able to continue their heavy work, whereas 78% (18/23) of the patients engaged in light physical work were able to keep working. None of the patients who were engaged in light physical work at T0 changed into heavy physical work at T1. In contrast, 5 patients who were engaged in heavy physical work at T0 changed into light physical work at T1. This results in a reduction of the proportion of VO patients engaged in heavy physical work from 56% to 23% and in light physical work from 44% to 35% one year after VO.

The relation between BMI and type of work is shown in Table 3. Patients with a BMI < 25 tend to be engaged more often (75%) in heavy physical work compared to those patients with a BMI > 25 (52%).

## 4. Discussion

The present study analyzing the workforce among a cohort of working-age VO patients was able to show that a large proportion (42%) did not return to work one year after treatment. Patients pursuing heavy physical work especially were no longer able to follow their occupation. Main factors associated with a sustained ATW were a low number of comorbidities (≤1) and a BMI > 25. However, nearly one third (27%) received a disability pension one year after treatment.

Baseline clinical characteristics in our cohort study were in line with other studies [1,3,7]. VO predominantly affected men (73%) in our cohort. Moreover, VO was mostly diagnosed in lumbar spine (67%). The majority of the cases were caused by *S. aureus* (32%). Unlike other studies, the severity and number of comorbidities were lower, probably due to the lower age range in our study.

Previous studies have shown VO as a severe disease with an overall mortality rate up to 20%, which appears to be particularly high in the first year after diagnosis [2,4,5,6]. In our cohort, the mortality rate was lower (6%), presumably due to the relatively young age and fewer comorbidities. Accordingly, other studies, which included primarily younger VO patients, also report a lower mortality. For instance, Dragsted et al. analyzed mortality in VO patients undergoing surgery in a cohort with a median age of 60 years and a one-year mortality of 6.5% [11]. Additionally, Kehrer et al. analyzed VO patients in a working-age population and reported a one-year mortality of 7% [13], again indicating that our cohort represents the typical course of VO.

An exclusive consideration of functional aspects (e.g., neurological deficits), mortality and recurrence in the treatment of VO is sufficient to reflect neither the complexity nor the consequences of the disease [6,10,12]. Further long-lasting negative effects, such as pain and reduction of workforce, even after healing, should also be considered [7,8,13]. To date, available studies on workforce after VO treatment are rare. Looking at these studies, there is a trend towards decreased ATW after VO and an increase in disability pension or retirement [13]. In a comparison of VO patients versus a reference working age population (20–57 years), Kehrer et al. showed that patients with VO were less likely to be part of the workforce before infection. Moreover, a fourth of the patients who were part of the workforce one year before VO did not return to workforce and 19% received disability pension during the two-year follow up [13]. In our cohort, the ATW one year after infection also decreased compared to baseline: nearly half of the cases (22/52) did not re-join the workforce and the proportion of patients on disability pension became 25%. A comparison of our results with the study by Kehrer et al. reveals a higher number of patients who did not return to work (42% vs. 27%) or received a disability pension (25% vs. 19%), in a one-year period after infection. One explanation for the higher rates in our study might be the higher age of inclusion (18–63 years) compared to Kehrer et al. (20–57 years). Presumably, we included more patients, who were more likely to receive a disability pension after this severe illness. In a systematic review, Rutges et al. showed that neurological deficit and recurrence occur in one third of cases after VO, respectively [10]. Among patients with VO, up to 35% never regain full physical function and this may partly explain the large proportion on disability pension after one year. However, we were not able to show an association between having neurological deficits at admission and the ATW, which might be explainable with the low rate (12%) of patients with neurological impairment in our cohort. However, a pre-operative neurological deficit had no significant influence on ATW and patients not working after VO tend to have a higher rate of neurological impairment (18% vs. 7%).

In their study, Kehrer et al. showed that capacity to work one year before VO positively correlated with the return to work (RTW). However, they were not able to identify any other patient- or disease-specific factors influencing RTW. In the past, several retrospective studies have found increasing numbers of comorbidities to be predictors for a poor outcome in VO [14,15,16,17]. These findings could be reproduced in our study as a higher number of comorbidities (≥2) was associated with the loss of ATW. Interestingly, patients with a BMI > 25 were more likely to regain their ATW. This finding is in line with several studies, which have linked overweight and obesity with a better outcome than normal weight in the context of different medical conditions, such as cardiovascular and pulmonary disease [18,19]. Moreover, septic overweight patients had a lower adjusted mortality, again supporting overweight as a protective factor for a positive outcome of a severe infection [20].

The results of our study revealed that the number of comorbidities (≥2) and BMI (>25) are important predictive factors for ATW.

In our study, 42% of the VO patients did not return to work one year after VO treatment, suggesting that VO is a debilitating condition comparable to a disability. Internationally, economic and social impacts related to disability have been analyzed from many aspects. For example, the Irish national disability authority gathering information about health, employment and poverty found that people with disabilities are 2.5 times less likely to be in work, leading to a significantly higher rate of poverty [21].

Among our study cohort, only 41% of the patients engaged in heavy physical work before VO were able to continue their work, whereas 78% of those patients engaged in light physical work before VO were able to keep working. Assuming that those patients who were engaged in heavy physical work were also those who earned less money, the effect of VO was much more burdensome in this group. A loss of workforce will therefore have a significantly stronger impact on patients engaged in heavy physical work than on those engaged in light physical work. It is likely that the latter population can better compensate for the loss of income and suffer less from the long-term consequences.

Interestingly, patients with a BMI < 25 tend to be engaged more often (75%) in heavy physical work. On the other hand, patients with a BMI > 25 seem more likely to carry out light physical work and can thus maintain their workforce more often. This might be an explanation as to why a BMI > 25 appears to be a protective factor to sustain ATW in our univariate analysis.

Additionally, the proportion of full-time working VO patients decreased from 73% to 31%. Only 10% of the VO patients were retrained and changed their profession in order to sustain their workforce. Thus, it is obvious that RTW is difficult, despite successful therapy indicating a strong impact of VO on the ATW. RTW requires not only intensive rehabilitation, but also special state reintegration programs [22].

Strikingly, VO patients working one year after treatment were more likely to be satisfied with their treatment in contrast to those not working. This finding suggests that ATW and patient satisfaction may be reduced, even in the case of a supposedly successful therapy. For this reason, not only the assessment of ATW but also patient satisfaction or Quality of Life (QoL) should be included in any evaluation of the treatment outcome.

The strengths of the current study include the study design with uniform one-year follow-up that yields more detailed and robust information, with strict inclusion criteria on the workforce of VO patients. To the best of our knowledge, this is the first study revealing the details on VO patients’ profession and their work time. By focusing on the ATW of VO patients to assess predictive factors for the workforce, our findings add important new clinically relevant information.

Limitations include a possible selection bias, as the study is a single-center study from a tertiary care hospital primarily treating multimorbid patients and/or patients with complicated case histories. Another limitation is the small number of analyzed patients (*n* = 52), whereas the only comparable study on this topic has a similar patient number (*n* = 48) [13]. Nevertheless, in the future additional studies with a higher number of working patients would be desirable to further confirm our results.

## 5. Conclusions

In conclusion, our study underlines that VO is a debilitating condition that affects patients’ ATW, especially working-age patients. VO is associated with long duration of sick leave and a high risk for disability pension and death. Our findings can already facilitate an estimation of a VO patients’ ATW after treatment. In the majority of cases, patients engaged in heavy physical work are more severely affected and are no longer able to keep their workforce. Generally, a BMI > 25 seems to be protective, since this subgroup of patients is more likely engaged in light physical work and is therefore more often able to maintain their workforce after VO. Only 10% of VO patients are retrained and change their profession. In our opinion, more support in retraining should be offered to VO patients after successful treatment to reduce the number of disabled pensions. These efforts could not only reduce the socio-economic burden for society, but could also improve patients’ treatment satisfaction.

## Figures and Tables

**Table 1:** Patient characteristics and clinical data at baseline of 52 patients at work and not at workforce one year after vertebral osteomyelitis (VO).

	All VO Patients 18–≤63 (*n* = 114)	VO Patients at Work T0 (*n* = 52, 19%)	VO Patients at Work T1 (*n* = 30, 58%)	VO Patients Not at Work T1 (*n* = 22, 42%)	*p*-Value *
**Age** (years) Median (interquartile range)	55 [48; 59]	55 [48; 59]	55 [48; 58]	56 [47; 61]	0.373
**Male** *n* (%)	83 (73)	42 (81)	24 (80)	18 (82)	0.869
**BMI** *n* (%)	98 (86)	45 (87)	24 (80)	21 (95)	0.023 *
Underweight	7 (7)	2 (4)	0 (0)	2 (10)	
Normal	31 (32)	11 (24)	4 (17)	7 (33)	
Overweight	28 (29)	18 (40)	14 (58)	4 (19)	
Obese	32 (33)	14 (31)	6 (25)	8 (38)	
**CCI** Median (interquartile range)	2 [1; 3]	1 [1; 2]	1 [0; 2]	2 [1; 3]	0.055
**Underlying Comorbidities**					
Diabetes *n* (%)	22 (19)	8 (15)	5 (17)	3 (14)	0.764
Malignancy *n* (%)	18 (16)	5 (10)	4 (13)	1 (5)	0.268
Rheumatic disease *n* (%)	9 (8)	3 (6)	0 (0)	3 (14)	0.020 *
Cardiac insufficiency *n* (%)	6 (5)	1 (2)	0 (0)	1 (5)	0.186
Chronic kidney disease *n* (%)	11 (10)	2 (4)	0 (0)	2 (9)	0.060
Alcohol abuse *n* (%)	14 (12)	4 (8)	2 (7)	2 (9)	0.747
Drug abuse *n* (%)	11 (10)	3 (6)	1 (3)	2 (9)	0.381
**Number of comorbidities**					0.007 *
0 *n* (%)	42 (37)	31 (60)	18 (60)	13 (59)	
1 *n* (%)	34 (30)	13 (25)	11 (37)	2 (9)	
≥2 *n* (%)	38 (33)	8 (15)	1 (3)	7 (32)	
**ASA**	106 (93)	49 (94)	27 (30)	22 (100)	
1 *n* (%)	9 (8)	6 (12)	4 (15)	2 (9)	
2 *n* (%)	40 (38)	28 (57)	17 (63)	11 (50)	
>3 *n* (%)	57 (54)	15 (31)	6 (22)	9 (41)	
**Bacteremia** *n* (%)	35 (31)	15 (29)	9 (30)	6 (27)	0.830
**Pathogen detected** *n* (%)	89 (78)	40 (77)	23 (77)	17 (77)	0.414
*S. aureus*	37 (32)	19 (37)	10 (33)	9 (41)	
CNS	20 (18)	10 (19)	4 (13)	6 (27)	
GN	7 (6)	1 (2)	1 (3)	0 (0)	
*Enterococcus* species	7 (6)	1 (2)	1 (3)	0 (0)	
Mycobacteria	4 (4)	2 (4)	1 (3)	1 (5)	
*Proprionibacterium* species	3 (3)	2 (4)	2 (7)	0 (0)	
Anaerobes	3 (3)	2 (4)	1 (3)	1 (5)	
*Streptococcus* species	3 (3)	1 (2)	1 (3)	0 (0)	
*Candida* species	2 (2)	1 (2)	1 (3)	1 (5)	
*Corynebacterium* species	1 (1)	1 (2)	1 (3)	0 (0)	
**ID Consultation** *n* (%)	99 (87)	46 (89)	25 (83)	21 (96)	0.155
**Manifestations** *n* (%)					
Endocarditis	5 (4)	1 (2)	0 (0)	1 (5)	0.186
Psoas Abscess	20 (18)	7 (13)	4 (13)	3 (14)	0.975
Empyema	44 (39)	22 (42)	13 (43)	9 (41)	0.861
**Spinal level**					0.405
Cervical *n* (%)	5 (4)	2 (4)	1 (3)	1 (5)	0.823
Thoracic *n* (%)	26 (23)	11 (21)	4 (13)	7 (32)	0.108
Lumbar *n* (%)	76 (67)	37 (71)	24 (80)	13 (59)	0.100
Multilevel *n* (%)	7 (6)	2 (4)	1 (3)	1 (5)	0.823
**Segments affected**					0.167
1 *n* (%)	89 (78)	42 (81)	26 (87)	16 (73)	
>1 *n* (%)	25 (22)	10 (19)	4 (13)	6 (27)	
**Neurological Deficit (Frankel)** *n* (%)	25 (22)	6 (12)	2 (7)	4 (18)	0.201
A	3 (3)	0 (0)	0 (0)	0 (0)	
B	3 (3)	1 (2)	0 (0)	1 (5)	
C	10 (9)	3 (6)	1 (3)	2 (9)	
D	9 (8)	2 (4)	1 (3)	1 (5)	
E	89 (78)	46 (88)	28 (93)	18 (82)	
**Vertebral Destruction (Eysel/Peters)** *n* (%)					0.423
1	13 (11)	9 (17)	5 (17)	4 (18)	
2	64 (56)	35 (67)	22 (73)	13 (59)	
3	35 (31)	8 (15)	3 (10)	5 (23)	
**Surgical Treatment** *n* (%)	99 (87)	45 (87)	24 (80)	21 (96)	0.087
**Recurrent disease** *n* (%)	11 (10)	6 (12)	3 (10)	3 (14)	0.687
**Physical work** (at T0)					0.120
Heavy *n* (%)		29 (56)	14 (47)	15 (68)	
Light *n* (%)		23 (44)	16 (53)	7 (32)	
**Treatment Satisfaction** (at T1)					0.020 *
1 *n* (%)		4 (8)	0 (0)	4 (18)	
2 *n* (%)		9 (17)	5 (17)	4 (18)	
3 *n* (%)		11 (21)	5 (17)	6 (27)	
4 *n* (%)		28 (54)	20 (67)	8 (36)	

* = *p*-value ≤ 0.05; *p*-value refers to the comparison of the VO patients at work and not at work at T1. BMI = Body mass index. CCI = Charlson comorbidity index. ASA = American Society of Anesthesiologists score. CNS = Coagulase-negative staphylococci. GN = Gram-negative bacteria. ID = Infectious diseases. One affected segment defines two vertebral bodies centering one disc space. All statistical tests are two-tailed.

**Table 2:** Development of workforce of patients before (T0) and one year after (T1) VO treatment (*n* = 52).

	Patients at T0 *n* (%)	Patients at T1 *n* (%)
at work	52 (100)	30 (58)
not at work	0 (0)	22 (42)
Disability pension	0 (0)	13 (25)
Heavy physical work	29 (56)	12 (23)
Light physical work	23 (44)	18 (35)
Full-time (100%)	38 (73)	16 (31)
Part-time (75–≤100%)	8 (15)	4 (8)
Part-time (50%)	4 (7)	3 (6)
Part-time (<50%) Retraining Sick leave	2 (4) n.a. n.a.	1 (2) 5 (10) 9 (17)

**Table 3:** Correlation between patient BMI and type of work.

	Patients BMI < 25 at T0 *n*(%)	Patients BMI > 25 at T0 *n*(%)	*p*-Value
Heavy physical work	9 (75)	17 (52)	0.158
Light physical work	3 (25)	16 (48)	

BMI = body mass index.

## Data Availability

Data available on request due to restrictions eg privacy or ethical. The data presented in this study are available on request from the corresponding author.
